# Metabo groups in response to micronutrient intervention: Pilot study

**DOI:** 10.1002/fsn3.1357

**Published:** 2019-12-19

**Authors:** Carolina Almeida Coelho‐Landell, Roberta Garcia Salomão, Maria Olimpia Ribeiro do Vale Almada, Mariana Giaretta Mathias, Roseli Borges Donega Toffano, Elaine Hillesheim, Tamiris Trevisan Barros, Joyce Moraes Camarneiro, José Simon Camelo‐Junior, José Cesar Rosa, Clarice Izumi, Érika Czernisz, Sofia Moco, Jim Kaput, Jacqueline Pontes Monteiro

**Affiliations:** ^1^ Department of Pediatrics and Department of Health Sciences Faculty of Medicine of Ribeirão Preto University of São Paulo São Paulo Brazil; ^2^ Department of Molecular and Cell Biology and Pathogenic Bioagents Protein Chemistry Center Medical School of Ribeirão Preto University of São Paulo Ribeirão Preto Brazil; ^3^ Nestlé Institute of Health Sciences Nestle Research EPFL Innovation Park Lausanne Switzerland; ^4^ Present address: Vydiant Sacramento CA USA

**Keywords:** genetic ancestry, intervention study, lipid profile, metabolomics, proteomics, Vitamin plasma levels

## Abstract

Micronutrients and their metabolites are cofactors in proteins involved in lipid metabolism. The present study was a subproject of the Harmonized Micronutrient Project (ClinTrials.gov # NCT01823744). Twenty participants were randomly selected from 136 children and adolescents that consumed a daily dose of 12 vitamins and 5 minerals supplementation for 6 weeks. The 20 individuals were divided into two pools of 10 individuals, according to their lipid profile at baseline (Pool 1 with lower triglycerides, LDL, and VLDL). The individuals were analyzed at baseline, after 6 weeks of daily supplementation, and after 6 weeks of a washout period in relation to anthropometric, body composition, food intake, lipid profile, micronutrient levels, and iTRAQ proteomic data. Genetic ancestry and its association with vitamin serum levels were also determined. After supplementation, LDL levels decreased while alpha‐tocopherol and pantothenic acid levels increased in pool 2; lipid profiles in pool 1 did not change but had higher plasma levels of pantothenic acid, pyridoxal, and pyridoxic acid. In pool 2, expression of some proteins increased, and expression of other ones decreased after intervention, while in pool 1, the same proteins responded inversely or did not change their levels. Plasma alpha‐tocopherol and Native American genetic ancestry explained a significant fraction of LDL plasma levels at baseline and in response to the intervention. After intervention, changes in expression of alpha‐1 antitrypsin, haptoglobin, Ig alpha‐1 chain C region, plasma protease C1 inhibitor, alpha‐1‐acid glycoprotein 1, fibrinogen alpha, beta, and gamma‐chain in individuals in pool 2 may be associated with levels of LDL and vitamin E. Vitamin E and Native American genetic ancestry may also be implicated in changes of vitamin E and LDL levels. The results of this pilot study must be validated in future studies with larger sample size or in in vitro studies.

## Graphical abstract

Changes in expression of alpha‐1 antitrypsin, haptoglobin, Ig alpha‐1 chain C region, plasma protease C1 inhibitor, alpha‐1‐acid glycoprotein 1, fibrinogen alpha, beta and gamma chain in response to a micronutrient intervention may be associated with levels of LDL and vitamin E in children and adolescents. Baseline vitamin E levels and Native American genetic ancestry may also be implicated in changes of vitamin E and LDL levels.

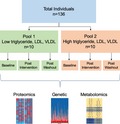

## 1 INTRODUCTION

Being overweight in childhood and adolescence may be associated with established predictors of cardiovascular disease including increased levels of glucose, triglycerides, total cholesterol, LDL cholesterol, and the low levels of HDL cholesterol (Alberti, Zimmet, & Shaw, 2006; Gröber‐Grätz et al., 2013; Quijada et al., 2008; Rosini, Moura, & Rosini, 2015; Tailor, Peeters, & Norat, 2010; Weiss & Kaufman, 2008). These risk factors are sensitive to nutritional intake. Balanced, nutrient‐dense diets can help achieve and maintain an adequate lipid, glycemic, and nutritional status profiles (Güngör, 2014).

Micronutrients and their metabolites are cofactors in enzymes including a subset involved in lipid metabolism (Al‐Attas et al. (2014); Kelishadi, Farajzadegan, & Bahreynian, 2014; Kelishadi et al., 2010). Almost all recommendations for micronutrient intake are based on the average in groups of individuals. In many cases, these recommendations are based on fasting levels in (presumably) healthy people and data for children and adolescents are sparse in many populations. We and others proposed to evaluate metabolic responses to acute challenges (e.g., oral glucose or mixed meal) or short‐term interventions (e.g., multiple micronutrient challenges) (Kaput & Morine, 2012; Kaput et al., 2014; Mathias et al., 2018; Ommen, Greef, & Ordovas, 2014; Pellis et al., 2012; Stroeve, Wietmarschen, & Kremer, 2015) to provide additional information about nutritional needs of individuals rather than just at baseline status. Metabolic responses are defined as changes in levels of not only the target metabolite or its surrogate (e.g., vitamin or lipoprotein) but also other biochemical variables (e.g., plasma proteins, micro RNAs, and other metabolites). Supplementing intake with a complex mixture of vitamins and minerals to an otherwise calorie‐sufficient diet improved metabolic health of Brazilian children and adolescents (Mathias et al., 2018).

The integration of these metabolic readouts provides a more comprehensive description of the physiological system and a more informative description of health. The ability to analyze different metabolites, proteins, RNA, or other blood molecules depends on the sensitivity of technologies, which may constrain the analysis. *Isobaric Tag for relative and absolute quantitation* (iTRAQ) allows for the discovery of blood or plasma proteins altered by nutritional intervention.

Genetic ancestry is also an important factor that can influence metabolite requirements in individuals and also affect population‐level‐derived averages. We (Mathias et al., 2018) and others (Kehdy et al., 2015; Rolim et al., 2016) have used admixture to better understand genetic and metabolite differences of individuals in subgroups of the Brazilian populations. We tested the relationship between an individual's genetic ancestry and micronutrient levels because the Brazilian population is highly admixed (Amerindians, European colonizers, African slaves, and more recent introgression due to immigration from other world regions, e.g., Asia). Linear regressions between ancestral components and baseline vitamin levels showed higher thiamine monophosphate (TMP) levels with higher European ancestry. Plasma vitamin B12 was negatively associated with increasing Native American ancestry. Finally, Native American ancestry was associated with lower baseline folate levels and greater response to the intervention (Mathias et al., (2018)). These results deserve further evaluation since vitamin levels may be implicated in reduction of LDL (Mathias et al., (2018)), an important predictor of cardiovascular disease.

We hypothesized that a metabolic group with a poor lipid profile would benefit most from micronutrient intervention and thus improve metabolic health through changes in expression of some proteins closely related to lipid metabolism. We also hypothesized that some improvements on vitamins and lipid levels could be associated with genetic ancestry.

The study aimed to: (a) evaluate the changes in proteomic profile, nutritional status, and vitamin serum levels after a micronutrient intervention in two lipid profile groups and (b) associate vitamin and lipid levels with genetic ancestry. The results of this pilot study must be validated in future studies with larger sample size or in vitro and experimental designs.

## 2 METHODS

### 2.1 Pilot study design and Population

This study was a subproject of the Harmonized Micronutrient Project (ClinTrials.gov # NCT01823744) that analyzed omics, biochemical, and nutritional status (Mathias et al., 2018) at baseline (time point or visit 1); after 6 weeks of daily supplementation of vitamins and minerals (time point or visit 2); and after 6 weeks of a washout period (time point or visit 3). Participants were healthy children and adolescents (ages 9–13) recruited from the west side of Ribeirão Preto (Brazil) in two county schools and one private school. For this specific pilot study, 20 participants were randomly selected from a sample size of 136 children and adolescents that were previously included following specific exclusion criteria: (a) one or more episodes of axillary temperature higher than 37°C within the 15 days preceding the data collection, (b) three or more episodes of liquid stools within the 24 hr before assessment, (c) current intake of vitamin or mineral supplement; dietary restrictions of any time, including weight‐loss interventions, and (d) history of chronic diseases; participation in another clinical trial within the 4 weeks preceding the study (Mathias et al., 2018). The 20 participants were divided into two metabolic groups according only to their lipid profiles: individuals in pool 1 (*n* = 10) had lower triglycerides, LDL, and VLDL levels, and individuals in pool 2 (*n* = 10) had higher triglycerides, LDL, and VLDL levels at baseline. The participants were evaluated by a pediatrician to determine their clinical conditions and pubertal stage, according to Tanner's criteria (Tanner, 1962) in visit 1, 2, and 3.

All participants received a daily supplement of 12 vitamins and 5 minerals in a commercial milk bar (Nestrovit^®^) (Table 1) for 5 days per week for 6 weeks. This product was chosen because it (a) was palatable (which would facilitate acceptance by participants), (b) had low amounts of calories (3 milk bars contains 75 calories), (c) has been commercially available in Switzerland since 1936 but never sold in Brazil, and (d) had a known and standard nutritional composition, all of which met the objectives of this study. Six of the authors individually monitored supplement intake at the beginning of each school period, and therefore, the compliance rate for the individuals in this substudy was 100%.

**Table 1:** Milk Bar Composition by tablets and comparisons with Dietary Reference Intakes (DRIs) and Upper Tolerable Intake Levels (UL)

Micronutrients	2 Milk bars	3 Milk bars	DRIs 9–13 years		UL 9–13 years	
			Boys	Girls	Boys	Girls
Vitamin A	534 µg	801 µg	600 µg	600 µg	1,700 µg	1,700 µg
Vitamin E	6.6 mg	9.9 mg	11 mg	11 mg	600 mg	600 mg
Folate	133.3 µg	200 µg	300 µg	300 µg	600 µg	600 µg
Vitamin B1	0.93 mg	1.4 mg	0.9 mg	0.9 mg	–	–
Vitamin B2	1.17 mg	1.76 mg	0.9 mg	0.9 mg	–	–
Niacin	12 mg	18 mg	12 mg	12 mg	20 mg	20 mg
Vitamin B6	1.33 mg	2 mg	1.0 mg	1.0 mg	60 mg	60 mg
Vitamin B12	0.73 µg	1.1 µg	1.8 µg	1.8 µg	–	–
Vitamin D3	3.4 µg	5.1 µg	5.0 µg	5.0 µg	50 µg	50 µg
Vitamin C	40 mg	60 mg	45 mg	45 mg	1,200 mg	1,200 mg
Biothine	13.3 µg	150 µg	20 µg	20 µg	–	–
Pantothenate	4 mg	6 mg	4 mg	4 mg	–	–
Calcium	191.3 mg	287 mg	1,300 mg	1,300 mg	2,500 mg	2,500 mg
Phosphorus	144.6 mg	217 mg	1,250 mg	1,250 mg	4,000 mg	4,000 mg
Iron	4.3 mg	6.5 mg	8 mg	8 mg	40 mg	40 mg
Magnesium	83.3 mg	125 mg	240 mg	240 mg	350 mg	350 mg
Zinc	5.3 mg	6 mg	8 mg	8 mg	23 mg	23 mg

Note

Comercial name: “Nestrovit”, brand: “Nestlé”. 2 milk bars (10 g): 51.3 kcal, 4.4 g of carbohydrate, 0.5 g of protein and 3.4 g of lipid. 3 milk bars (15 g): 77 kcal, 6.7 g of carbohydrate, 0.8 g of protein and 5.2 g of lipid.

### 2.2 Blood collection and Laboratory analyses

Blood samples were taken after 12 hr fasting in EDTA tubes for metabolomics and proteomics, in PAXgene tubes for DNA analysis, and separately in ACD tubes for clinical biochemistry. After centrifugation, plasma was removed and 100 μl was frozen for iTRAQ proteomic analysis. Clinical biochemistry, micronutrient, dietary intake, genotype analyses, and plasma vitamin response were described previously (Mathias et al., 2018).

For the iTRAQ proteomic analysis, 6 sample pools were made: pool 1 at Visit 1 (P1V1), Visit 2 (P1V2), and Visit 3 (P1V3), and pool 2 at Visit 1 (P2V1), Visit 2 (P2V2), and Visit 3 (P2V3). These 6 pooled plasma samples were dilapidated and depleted of the most abundant plasma proteins using the Proteopep Immunoaffinity Albumin and IgG depletion kit (Sigma^®^), according to manufacturer's protocol. Total proteins of each pool were quantified by the method of Bradford (1976). After preparation, the samples were submitted to enzymatic hydrolysis with trypsin. Tryptic peptides from each pool were labeled with isobaric tag for relative and absolute quantitation using the iTRAQ 8‐plex kit (AB Sciex^®^) according to the manufacturer's instructions. Each peptide solution was labeled at room temperature for 2 hr with one iTRAQ reagent vial (mass tag 113 (P1V1), 114 (P1V2), 115 (P1V3), 116 (P2V1), 117(P2V2), and 118 (P2V3)). iTRAQ reagent‐labeled samples were combined into one tube and then dried to completeness. After lyophilization, the fractions were dissolved in 25 μl of 0.1 M ammonium formate and 5% (v/v) acetonitrile and taken for liquid chromatography (LC)–mass spectrometry (MS) analysis. The LC‐MS was composed of an ultra‐HPLC NanoAcquity (Waters) coupled to an ESI‐Q‐TOF‐MS instrument. MS/MS spectra were extracted with Thermo Scientific Xtract software. The generated data were analyzed using Mascot^®^ (Matrix Science) by Proteome Discoverer (v1.3, Thermo Scientific) software. Scaffold Q + S (Proteome Software Inc.) was used to sum the spectral counts.

Vitamin measurements were previously analyzed (Mathias et al., 2018). In this study, vitamin medians (minimum–maximum) were used for individuals in pool 1 and pool 2.

### 2.3 Anthropometric and body composition data

Height and weight were measured immediately following blood collection (Jelliffe, 1968). Body mass index (BMI) was used as criteria for weight status (World Health Organization, 2007). Waist circumference was measured at the level of the imaginary horizontal line in the middle region between the last rib and the iliac crest (Heyward & Stolarczyk, 1996). Body composition analysis was performed by bioelectrical impedance analysis, according to Lukaski, Bolonchuk, and Hall (1986) immediately following the blood draw and before breakfast.

### 2.4 Food intake data

The usual dietary intake was assessed by a food frequency questionnaire (FFQ) of the preceding month using a previously validated questionnaire for Brazil children at each of the three study time points (Fumagalli, Monteiro, & Sartorelli, 2008). The food frequency questionnaire was applied using pictures from Monteiro and Chiarello (2007) depicting three portion sizes (small, medium, and large) of usual Brazilian foods. DietWin Profissional^®^ software version 2011 (Dietwin Software de Nutrição, 2018) was used for analyzing the nutritive value of the foods regarding energy, carbohydrate, protein, and lipids.

### 2.5 Genetic ancestry

Genetic analysis and ancestry determination were previously analyzed and reported in Mathias et al. (2018). Average ancestry data for individuals in each pool were calculated by summing percent of individual ancestry and dividing by 10.

### 2.6 Statistical analysis

SPSS 20.0 program^®^ was used to analyze metabolic and nutritional data. Mann–Whitney and Student's *t* tests were used to compare two pools. For longitudinal analysis, ANOVA for repeated measurements was used adjusting by Bonferroni test. Chi‐square test was used to compare proportions. The intensities found in iTRAQ analyses were expressed by fold change of the pool and not of the individual. The fold change of the pool is the ratio between the quantitative values of a given protein between baseline and postintervention. Proteins with fold change ≥1.20 or ≤0.80 were considered as differentially expressed proteins, as described in other studies (Duthie, Osborne, & Foster, 2007; Moulder et al., 2010; Seshi, 2006; Unwin et al., 2006). Simple and multiple variate linear regression approaches were used to test associations between the ancestral components and lipid and vitamin levels in the 20 participants. Statistical significance was considered when *p* < .05.

## 3 RESULTS

Twenty individuals from a larger cohort were classified according to their lipid profile in two pools (ool 1 with lower triglycerides, LDL, and VLDL than pool 2). Tables 2, 3, 4, 5 present variables statistically different or close to statistical significance between pool 1 (*n* = 10 individuals) and ool 2 (*n* = 10 individuals). pool 1 had more females (70%) and less males compared with pool 2 (female = 30%; male = 70%), which almost reach statistical significance (*p* = .074). Age was not different among pools (pool 1 was 11.4 ± 1.2 vs. 11.8 ± 0.8 years old, *p* = .481).

**Table 2:** Comparison of anthropometric measurements, body composition profile, and gender at baseline between the pools

Variable	pool 1 (*n* = 10)	pool 2 (*n* = 10)	*p* value
BMI (kg/m^2^) V1	20.5 (14.5–31.0)	23.6 (16.0–41.3)	.138
WC (cm) V1	69.4 (55.5–107.2)	81.4 (43.6–127.4)	.250
LM (% weight) V1	75.3 (62.3–79.9)	68.9 (61.3–79.9)	.188
FM (% weight) V1	24.7 (20.1–37.7)	31.0 (20.1–38.7)	.206
Percentage of Females (%)	70.0	30.0	.074

Note

Results are presented as median (minimum–maximum) in visit 1 (V1). Student's *t* test was applied for comparison between pools. Chi‐square test was used to compare gender proportions.

Abbreviations: %, percentage; BMI, body mass index; cm, centimeter; dl, deciliter; FM, fat mass by bioimpedance; kg, kilogram; LM, lean mass by bioimpedance; m, meter; mg, milligram; V1, visit 1; WC, waist circumference.

**Table 3:** Comparison of energy and macronutrients intake between pools

Variable	pool 1 (*n* = 10)	pool 2 (*n* = 10)	*p* value
Mean Energy (kcal)	1,725.6 ± 395.0	1,852.8 ± 578.4	.573
Mean CHO (g)	235.1 ± 51.5	254.5 ± 97.1	.584
Mean LIP (g)	57.6 ± 16.2	61.7 ± 17.3	.593
Mean PTN (g)	66.6 ± 23.1	70.0 ± 13.6	.692

Note

Results are presented as mean ± standard deviation, according to average of the three visits. Student's *t* test was applied for comparison between pools.

Abbreviations: CHO, carbohydrates intake; FFQ, food frequency questionnaire; g, grams; Kcal, kilocalorie; LIP, lipid intake; PTN, protein intake.

**Table 4:** Comparison of lipid profile between the pools and throughout the study

Variable	pool 1 (*n* = 10)	pool 2 (*n* = 10)	*p* value
TC (mg/dl) V1	155.5 (119.0–210.0)	201.0 (112.0–240.0)	.105
TC (mg/dl) V2	148.5 (108.0–193.0)	171.5 (119.0–202.0)	.165
TC (mg/dl) V3	144.0 (119.0–212.0)	178.0 (96.0–214.0)	.063
Mean TC (mg/dl) (V1, V2, V3)	150.0 (118.0–205.0)	190.2 (109.0–208.7)	.089
TG (mg/dl) V1	70.5 (34.0–100.0)	98.0 (73.0–173.0)	**.011**
TG (mg/dl) V2	60.0 (33.0–119.0)	134.5 (101.0–385.0)	**<.001**
TG (mg/dl) V3	58.5 (40.0–104.0)	128.0 (25.0–206.0)	.063
Mean TG (mg/dl) (V1, V2, V3)	63.3 (39.3–103.7)	133.7 (75.0–220.3)	**<.001**
VLDL (mg/dl) V1	14.5 (7.0–20.0)	19.5 (15.0–35.0)	**.011**
VLDL (mg/dl) V2	12.0 (7.0–24.0)	27.0 (20.0–77.0)	**.000**
VLDL (mg/dl) V3	11.5 (8.0–21.0)	26.0 (5.0–41.0)	**.035**
Mean VLDL (mg/dl) (V1, V2, V3)	12.7 (8.0–21.0)	26.7 (15.7–44.0)	**<.001**
LDL (mg/dl) V1	91.0 (67.0–128.0)	130.5 (62.0–179.0)^a^	**.042**
LDL (mg/dl) V2	85.5 (59.0–112.0)	95.5 (27.0–137.0)^a^	.393
LDL (mg/dl) V3	83.0 (64.0–135.0)	101.5 (54.0–141.0)	.052
Mean LDL (mg/dl)	85.5 (66.7–124.0)	114.5 (47.7–152.3)	.143
HDL (mg/dl) V1	46.5 (33.0–67.0)	42.0 (28.0–66.0)	.280
HDL (mg/dl) V2	51.0 (39.0–77.0)	33.0 (26.0–68.0)	**.035**
HDL (mg/dl) V3	47.5 (31.0–66.0)	40.0 (26.0–60.0)	.218
Mean HDL (mg/dl)	48.5 (35.7–70.0)	38.7 (29.3–64.7)	.123

Note

Results are presented as median (minimum–maximum) according to study visit. Mann–Whitney test was applied for comparison between pools (*p* values < .05 are in bold). Longitudinal analysis by pairwise comparisons adjusted for Bonferroni.

Abbreviations: dl, deciliter; HDL, HDL cholesterol; LDL, LDL cholesterol; mg, milligram; TC, total serum cholesterol; TG, triglycerides; V1, visit 1; V2, visit 2; V3, visit 3; VLDL, VLDL cholesterol.

a Decreased from V1 to V2 (*p* < .05).

**Table 5:** Statistically different vitamins between pools and throughout the study

Variable	pool 1 (*n* = 10)	pool 2 (*n* = 10)	*p* value
α‐tocopherol (Vit E) V1 (µg/ml)	5.5 (3.3–7.2)	7.0 (3.2–8.3)^a^	.123
α‐tocopherol (Vit E) V2 (µg/ml)	5.6 (3.1–7.5)	8.0 (3.1–10.1)^a^	.063
γ‐tocopherol (Vit E) V1(µg/ml)	0.6 (0.3–1.2)	0.9 (0.6–1.1)	**.043**
Retinol (Vit A) V1 (µg/ml)	0.3 (0.2–0.4)	0.4 (0.3–0.5)	**.035**
Retinol (Vit A) V2 (µg/ml)	0.3 (0.2–0.4)	0.4 (0.3–0.6)	**.015**
5–Methyl–tetrahydrofolic acid (Vit B9) Mean (nmol/L)	15.0 (7.8–34.3)	26.4 (12.7–45.3)	**.035**
Thiamine monophosphate (Vit B1) V1 (nmol/L)	12.0 (7.0–21.6)	7.3 (5.0–14.0)	**.017**
Nicotinamide (Vit B3) V3 (nmol/L)	311.0 (244.0–404.0)	381.0 (277.0–627.0)	**.007**
Pantothenic Acid (Vit B5) V1 (nmol/L)	216.0 (186.0–416.0)^a^	215.0 (131.0–299.0)^a^	.684
Pantothenic Acid (Vit B5) V2 (nmol/L)	411.0 (225.0–559.0)^a^, ^b^	402.0 (244.0–516.0)^a^, ^b^	.912
Pantothenic Acid (Vit B5) V3 (nmol/L)	233.5 (140.0–480.0)^b^	231.0 (153.0–290.0)^b^	.684
Pyridoxal (Vit B6) V1 (nmol/L)	9.6 (3.9–12.4)^a^	9.5 (4.8–12.3)	.780
Pyridoxal (Vit B6) V2 (nmol/L)	13.1 (9.5–19.2)^a^, ^b^	10.4 (6.7–19.0)	.278
Pyridoxal (Vit B6) V3 (nmol/L)	7.7 (5.4–14.4)^b^	8.3 (4.0–19.6)	.684
Pyridoxic Acid (Vit B6) V1 (nmol/L)	18.3 (5.0–29.1)^b^	17.0 (10.3–42.7)	.853
Pyridoxic Acid (Vit B6) V2 (nmol/L)	29.7 (14.5–65.3)^b^	27.5 (13.1–56.1)	.579
Cobalamin (Vit B12) V1(pg/ml)	604.0 (355.0–1,539.0)	393.0 (227.0–843.0)	**.015**
Cobalamin (Vit B12) V2 (pg/ml)	604.0 (470.0–978.0)	402.5 (144.0–1,474.0)	**.043**
Cobalamin (Vit B12) V3 (pg/ml)	549.5 (253.0–1,207.0)	373.0 (294.0–538.0)	**.012**
Cobalamin (Vit B12) Mean (pg/ml)	595.0 (368.7–1,241.3)	394.2 (274.5–850.5)	**.035**

Note

Results are presented as median (minimum–maximum), according to study visit. Mann–Whitney test was applied for comparison between pools (*p* values < .05 are in bold). Longitudinal analysis by pairwise comparisons adjusted for Bonferroni.

a Increased from V1 to V2 (*p* < .05).

b Decreased from V2 to V3 (*p* < .05).

At baseline, pool 1 had lower gamma‐tocopherol (a form of vitamin E), retinol (vitamin A), and higher TMP (thiamine monophosphate, a form of vitamin B1) and vitamin B12 when compared to pool 2. At baseline, average nutrient intake, anthropometric measurements, and body composition did not differ between the pools (Tables 2 and 3) and also did not vary throughout the study in individuals of either pool (*p* > .05 for all parameters, data not shown). The lipid profile improved only in individuals in pool 2 with a decrease in LDL from V1 to V2 (Table 4). Although many vitamins increased from V1 to V2 after intervention and decreased from V2 to V3 (after wash out), only pantothenic acid (vitamin B5), pyridoxal (a form of vitamin B6), and pyridoxic acid (a catabolic product of vitamin B6) plasma levels in pool 1 and alpha‐tocopherol (a form of vitamin E) and pantothenic acid in pool 2 reached statistical significance (Table 5).

Twenty plasma proteins were identified by proteomic analysis that changed expression after micronutrient supplementation in at least one of the pools, and 18 presented fold change ratio ≥1.20 or ≤0.80. In addition, most of the identified proteins had different levels between the pools after the intervention (i.e., the same protein had increased expression in a pool and had decreased or unchanged expression in the other pool after the supplementation) (Table 6).

**Table 6:** Expression of the proteins in pool samples* identified by iTRAQ proteomic analysis

Identified Protein	Protein_ID	Fold Change pool 1			Fold Change pool 2		
		V2/V1	V3/V2	V3/V1	V2/V1	V3/V2	V3/V1
Alpha‐1‐acid glycoprotein 1	A1AG1_HUMAN	**1.92**	**0.52**	**1.24**	**0.65**	**1.24**	1.00
Alpha‐1‐antitrypsin	A1AT_HUMAN	1.00	**1.24**	**1.24**	**1.24**	1.00	1.00
Alpha‐2‐HS‐glycoprotein	FETUA_HUMAN	**1.24**	**0.52**	0.81	0.81	**1.54**	1.00
Alpha‐2‐macroglobulin	A2MG_HUMAN	0.81	**1.54**	1.00	1.00	1.00	1.00
Apolipoprotein A‐I	APOA1_HUMAN	0.81	0.81	0.81	1.00	0.81	1.00
Apolipoprotein A‐IV	APOA4_HUMAN	**0.65**	**1.54**	0.96	0.96	0.81	1.00
Apolipoprotein B‐100	APOB_HUMAN	1.01	**1.54**	**1.56**	0.81	**1.40**	1.19
Ceruloplasmin	CERU_HUMAN	**1.24**	**0.54**	**0.65**	**1.66**	0.92	**1.54**
Complement C3	CO3_HUMAN	0.81	**1.24**	1.00	1.00	**1.24**	**1.24**
Complement C4‐A	CO4A_HUMAN(+1)	1.00	**1.24**	**1.24**	0.81	**1.24**	1.00
Fibrinogen alpha chain	FIBA_HUMAN	**1.54**	**0.65**	1.00	**0.65**	**1.54**	1.00
Fibrinogen beta chain	FIBB_HUMAN	**1.24**	1.00	**1.24**	**0.65**	**1.54**	**1.24**
Fibrinogen gamma‐chain	FIBG_HUMAN	**1.54**	**0.65**	1.00	**0.65**	**1.92**	**1.24**
Haptoglobin	HPT_HUMAN	**0.65**	**1.71**	**1.24**	**1.54**	0.81	**1.24**
Ig alpha‐1 chain C region	IGHA1_HUMAN	**0.58**	**1.54**	1.00	**1.54**	0.92	**1.20**
Ig mu chain C region	IGHM_HUMAN	1.00	0.94	1.00	0.81	**1.24**	1.00
Plasma protease C1 inhibitor	IC1_HUMAN	1.00	**1.33**	**1.51**	**1.25**	**0.75**	1.00
Serotransferrin	TRFE_HUMAN	**0.52**	**1.54**	1.00	1.00	0.81	1.00
Serum albumin	ALBU_HUMAN	0.81	0.81	0.81	1.00	1.01	1.00
Vitamin D‐binding protein	VTDB_HUMAN	**0.65**	**0.65**	**0.42**	0.81	1.00	1.00

Note

V1, visit 1; V2, visit 2; V3, visit 3.

* Fold change ratio ≥ 1.20 or ≤0.80 are in bold and represent the differentially expressed proteins (see statistical analysis section).

Average genetic admixture differed between pools with a higher percentage of Native American ancestry in pool 2 compared to a higher percentage of ancestry from Europe in pool 1 (Table 7). African genetic ancestry was greater in pool 2 although the difference did not reach statistical significance. However, simple linear regression analysis applied to all subjects (*n* = 20) shows that genetic ancestry alone could not explain statistically different vitamin levels at baseline (Table 8).

**Table 7:** Comparison of genetic ancestry frequency between pools*

Variable	pool 1 (*n* = 10)	pool 2 (*n* = 10)	*p* value
African (%)	16.8 (4.8–58.6)	35.1 (10.4–96.8)	.06
Europe (%)	71.3 (17–89.9)	29.1 (0–69)	.004
Native America (%)	7.1 (0–23.7)	14 (3.1–43.8)	.031

* Participants were genetically admixed.

**Table 8:** Baseline vitamins statistically different between pools and their association with percentages of genetic ancestry

Variables	Gamma‐tocopherol	Retinol	TMP	Vit B12
		*r*; *R* ^2^; *p* value*		
African	.21; .05; .38	.24; .06; .33	−.37; .14; .13	−.30; .09; .22
Europe	−.27; .07; .27	−.27; .07; .28	.33; .11; .18	.27; .07; .27
America	.17; .03; .48	.12; .02; .62	.06; .00; .82	.01; .00; .96

Abbreviation: TMP, thiamine monophosphate.

* Simple linear regression analysis; *p* value according to ANOVA; *r* = Pearson correlation; *R* ^2^ = R Square.

Multiple regression analysis was used to find associations among metabolite levels in response to the intervention using alpha‐tocopherol and pantothenic acid (whose plasma levels changed after consumption of micronutrients), genetic ancestry, and sex. The small sample size in each pool (*n* = 10) required that all subjects (*n* = 20) be included in the analysis and just statistically significant results are presented. An increase in the percentage of Native America genetic ancestry and differences in sex can, together, predict 33% of alpha‐tocopherol plasma‐level variation in V2 (*r* = .64; *R* ^2^ = .41; adjusted *R* ^2^ = .33; ANOVA *p* = .01) as well as predict 29% of the fold change variation for alpha‐tocopherol from visit 1 to visit 2 (*r* = .62; *R* ^2^ = .38; adjusted *R* ^2^ = .29; ANOVA *p* = .007). Regression analysis did not find any association between the percentage of Native America ancestry and sex with plasma pantothenic acid in V1, V2, or for the fold change (V2–V1). Statistically significant associations were also not found between the percentage of European ancestry and sex with plasma alpha‐tocopherol or pantothenic acid in V1, V2, and for fold change (V2–V1).

Plasma alpha‐tocopherol and differences in sex in combination also predicted 32% of LDL plasma levels variation in V1 (*r* = .62; *R* ^2^ = .39; adjusted *R* ^2^ = .32; ANOVA *p* = .015) and 11% of LDL plasma levels variation in V2 (*r* = .45; *R* ^2^ = .20; adjusted *R* ^2^ = .11; ANOVA *p* = .05) using multiple regression analysis. The fold change for alpha‐tocopherol (V2–V1) and sex predicted 31% of fold change variation of LDL (*r* = .62; *R* ^2^ = .39; adjusted *R* ^2^ = .31; ANOVA *p* = .02). Plasma pantothenic acid fold change and differences in sex can predict 34% of the variation in fold change for LDL (*r* = .64; *R* ^2^ = .41; adjusted *R* ^2^ = .34; ANOVA *p* = .012).

Genetic ancestry from Native Americans and differences in sex predicted 43% of changes in LDL plasma levels (*r* = .71; *R* ^2^ = .49; adjusted *R* ^2^ = .43; ANOVA *p* = .006), while genetic ancestry from Europe and differences in sex predicted 30% of the variation in LDL plasma levels (*r* = .62; *R* ^2^ = .38; adjusted *R* ^2^ = .30; ANOVA *p* = .02).

We tested whether pantothenic acid, alpha‐tocopherol, sex, and genetic ancestry could together predict baseline LDL and after intervention. The fold change in plasma alpha‐tocopherol, fold change in plasma pantothenic acid, differences in sex, and the percentage of American and Europeans ancestry explained 39% of the variation in LDL levels (*r* = .75; *R* ^2^ = .56; adjusted *R* ^2^ = .39; ANOVA *p* = .04). We could not link genetic ancestry with proteomics because the samples were pooled.

## 4 DISCUSSION

iTRAQ methodology was used to identify plasma proteins altered by a multiple micronutrient intervention. Pooled analysis was done based on lipid profiles because of the required volume of sample needed for this technology. Although both pools were similar regarding age, food intake, and nutritional status, they differed in sex (although not statistically), certain plasma vitamins, and the proteins identified in this study. Individuals in pool 1 had lower triglyceride, LDL, VLDL, gamma‐tocopherol, retinol, and higher TMP and vitamin B12 when compared to individuals in pool 2. After supplementation, LDL levels of individuals in pool 2 decreased and also improved plasma levels of alpha‐tocopherol and pantothenic acid. pool 1 did not change lipid profile but had improvements in pantothenic acid, pyridoxal, and pyridoxic acid levels. These differences could not be explained by food intake, body composition, or nutritional status (which did not change throughout the study).

Proteomic analysis identified twenty plasma proteins whose levels varied between pools. Their main metabolic functions included lipid and glucose metabolism, transport/metabolism of vitamins and minerals, immune system function, blood clotting, and acute phase reactions (Bisoendial et al., 2015; Calder et al., 2013; Campenhout, Campenhout, & Lagrou, 2003; Carter & Worwood, 2007; Clerc et al., 2016; Dabrowska, Tarach, Wojtysiak‐Duma, & Duma, 2015; Davis, Mejia, & Lu, 2008; Engström, Hedblad, & Janzon, 2007; Gomme & Bertolini, 2004; Gruys, Toussaint, & Niewold, 2005; Hovland et al., 2015; Ix et al., 2006; Jenkins, Best, & Klein, 2004; Kohan, Wang, & Lo, 2015; Lee et al., 2010; Luo, Lei, & Sun, 2015; Musci, Polticelli, & Bonaccorsi di Patti, 2014; Ortiz, Salica, & Chuluyan, 2014; Rehman, Ahsan, & Khan, 2013; Sitar, Aydin, & Cakatay, 2013; Tesseromatis, Alevizou, & Tigka, 2011; Toonen et al., 2016; UniProt, 2016, 2017a, 2017b; Walldius & Jungner, 2004; Wang et al., 2015; Wu & Lyons, 2011). The levels of most of these proteins were altered after the micronutrient supplementation based on the observed fold changes.

In pool 2, expression of alpha‐1 antitrypsin, haptoglobin, Ig alpha‐1 chain C region, and plasma protease C1 inhibitor increased. These changes may be associated with the improvements in plasma LDL. The above proteins have been shown to be associated with positive physiological effects in lipid/glucose metabolism, micronutrients transport/metabolism, and in the immune system (Carter & Worwood, 2007; Davis et al., 2008; Toonen et al., 2016; UniProt, 2016, 2017a). In pool 2, expression of alpha‐1‐acid glycoprotein 1 and fibrinogen alpha‐, beta‐, and gamma‐chains decreased in response to the intervention. Alpha‐1‐acid glycoprotein 1 is a positive acute phase plasma protein (Luo et al., 2015; Tesseromatis et al., 2011), and high fibrinogen (Gruys et al., 2005) levels were positively associated with atherothrombotic disease (Aleman, Walton, & Byrnes, 2014; Perl et al., 2016; Poredoš & Ježovnik, 2015). A decrease in levels of these markers after intervention may benefit individuals in pool 2. Individuals in pool 1 did not show any improvements in lipid profile, and the analyzed proteins responded inversely or did not change their levels.

Multiple linear regression analysis applied to all subjects (*n* = 20) showed that sex and plasma alpha‐tocopherol predicted 32% of LDL plasma variation at baseline and 11% of LDL plasma levels variation in V2. Sex and fold change in alpha‐tocopherol and fold change in pantothenic acid plasma levels explained 31% and 34% of the fold change variation in plasma LDL levels, respectively. In addition, the percentage of America genetic ancestry and differences in sex could together predict 33% of alpha‐tocopherol plasma levels variation in V2, as well as 29% in fold change variation of alpha‐tocopherol that was found between visit 1 to visit 2. This is the first study showing a possible association between American genetic ancestry and vitamin E in children and adolescents. The role of vitamin E as an antioxidant is well known, but it also contributes to anti‐inflammatory responses through interleukin‐4, interleukin 8, TNF‐α, and inhibition of lipopolysaccharide secretion (Wu, Liu, & Ng, 2008). Moreover, vitamin E may protect against cardiovascular disease, improve lipid profiles, and reduce LDL oxidation (Burdeos, Nakagawa, & Kimura, 2012; Daud et al., 2013; Heng et al., 2013; Qureshi, Salser, & Parmar, 2001; Wu et al., 2008). Micronutrients, including pantothenic acid (vitamin B5), play an important role in lipid metabolism (Al‐Attas et al., 2014; Evans et al., 2014; Hadjistavri et al., 2010; Heng et al., 2013; Kelishadi et al., 2014, 2010), which supports the changes in LDL metabolism in the pool 2.

Differences in sex and genetic ancestry from Americans and from Europeans predicted 43% and 30% of fold change LDL plasma levels variation. Others have found association between American and Europeans genetic ancestry with LDL, even after adjusting for interactions with vitamin E (Dumitrescu et al., 2012, 2010).

The present pilot study found that the fold change in plasma alpha‐tocopherol, fold change in plasma pantothenic acid, differences in sex, and the percentage of American and Europeans ancestry explained 39% of the variation in fold change for LDL, corroborating some studies (Burdeos et al., 2012; Daud et al., 2013; Dumitrescu et al., 2012, 2010; Evans et al., 2014; Heng et al., 2013; Qureshi et al., 2001; Wu et al., 2008). To our knowledge, this is the first study that found these variables explained variation in LDL plasma levels after micronutrient supplementation.

This study has some limitations. Samples for pooling were selected based on differences in lipid profiles and were few in number. In addition, pooling eliminated the possibility of analyzing samples individually or testing the association of vitamin levels, proteomic data, and ancestry. These experimental choices were due to the high cost and time for this procedure. However, the use of pooling samples has been successfully used in several studies of proteomic analysis (Karp & Lilley, 2009; Kaur, Rizk, & Ibrahim, 2012; Weinkauf, Hiddemann, & Dreyling, 2006).

## 5 CONCLUSIONS

Ten individuals with similar high lipid profile at baseline responded positively (i.e., decreased LDL) to the intervention and also had increased alpha‐tocopherol and pantothenic acid levels. Changes after the intervention in the level of alpha‐, beta‐, and gamma‐fibrinogen chains, haptoglobin, Ig alpha‐1 chain C region, plasma protease C1 inhibitor, alpha‐2‐HS‐glycoprotein, alpha‐1 antitrypsin, and alpha‐1‐acid glycoprotein1 may be associated with changes of plasma LDL. Many of the proteins differed inversely between individuals in each of the pools, that is, while a protein had increased expression in one pool, the same protein had decreased or unchanged expression in the other pool. These results were consistent with the emerging awareness that individuals differ in response to the same nutritional intervention. The use of pools allowed for the identification of proteins correlated with changes in LDL levels using iTRAQ methodology. In addition, differences in sex, plasma alpha‐tocopherol, plasma pantothenic acid, and genetic ancestry directly or indirectly predicted LDL plasma levels in the total sample. The results of this pilot study must be validated in future studies in vitro, with animal models, and in human studies with larger sample sizes.

## CONFLICT OF INTEREST

Funding was provided by the Nestle Institute of Health Sciences (Lausanne, Switzerland) (contract reference RDHS 000054). Sofia Moco and Jim Kaput were employees at Nestlé Institute of Health Sciences and participated in the experimental design of the study and in writing the final manuscript, as well as other authors. All other authors declare no conflict of interest.

## ETHICAL APPROVAL

The study conforms to the Declaration of Helsinki for human subjects. The study's protocols and procedures were ethically reviewed and approved by the Research Ethics Committee of Clinical Hospital of Ribeirão Preto Medical School, University of São Paulo (HCRP Process Nº 14255/2010) and by National Commission for Ethics Research (CAAE Case No. 00969412.6. 0000.5440).

## INFORMED CONSENT

Written informed consent was obtained from all study participants. Each participant signed an assent form, and their parents (or legal guardians) signed a consent form prior to participation in this study.

## TRANSPARENCY DECLARATION

The lead author affirms that this manuscript is an honest, accurate, and transparent account of the study being reported. The reporting of this work is compliant with STROBE guidelines. The lead author affirms that no important aspects of the study have been omitted and that any discrepancies from the study as planned (ClinTrials.gov # NCT01823744) have been explained.
